# Spectrum of low-density lipoprotein receptor (*LDLR*) mutations in a cohort of Sri Lankan patients with familial hypercholesterolemia – a preliminary report

**DOI:** 10.1186/s12944-018-0763-z

**Published:** 2018-05-02

**Authors:** C. S. Paththinige, J. R. D. K. Rajapakse, G. R. Constantine, K. P. Sem, R. R. Singaraja, R. W. Jayasekara, V. H. W. Dissanayake

**Affiliations:** 1 Human Genetics Unit, Faculty of Medicine, University of Colombo, Kynsey Road, Colombo, 00800 Sri Lanka; 2 Faculty of Medicine and Allied Sciences, Rajarata University of Sri Lanka, Anuradhapura, Sri Lanka; 3 Department of Clinical Medicine, Faculty of Medicine, University of Colombo, Colombo, Sri Lanka; 4 Translational Laboratory in Genetic Medicine, Agency for Science Technology and Research, National University of Singapore, Singapore, Singapore

**Keywords:** Familial hypercholesterolemia, *LDLR* gene, Single nucleotide variants (SNV)

## Abstract

**Background:**

Hypercholesterolemia is a major determinant of cardiovascular disease-associated morbidity and mortality. Mutations in the LDL-receptor (*LDLR*) gene are implicated in the majority of the cases with familial hypercholesterolemia (FH). However, the spectrum of mutations in the *LDLR* gene in Sri Lankan patients has not been investigated. The objective of this study was to report the frequency and spectrum of variants in *LDLR* in a cohort of Sri Lankan patients with FH.

**Methods:**

A series of consecutive patients with FH, diagnosed according to Modified Simon Broome criteria or Dutch Lipid Clinic Network criteria at the University Medical Unit, Colombo, were recruited. Clinical data was recorded. DNA was extracted from peripheral blood samples. The *LDLR* gene was screened for genetic variants by Sanger sequencing.

**Results:**

A total of 27 patients [13 (48%) males, 14 (52%) females; age range 24–73 years] were tested. Clinical features found among these 27 patients were: xanthelasma in 5 (18.5%), corneal arcus in 1 (3.7%), coronary artery disease (CAD) in 10 (37%), and a family history of hypercholesterolemia and/or CAD in 24 (88.9%) patients. In the entire cohort, mean total cholesterol was 356.8 mg/dl (±66.4) and mean LDL-cholesterol was 250.3 mg/dl (±67.7). Sanger sequencing of the 27 patients resulted in the identification of known pathogenic missense mutations in 5 (18.5%) patients. Four were heterozygotes for 1 mutation each. They were c.682G > C in 2 patients, c.1720C > A in 1 patient, and c.1855 T > A in 1 patient. One patient with severe FH phenotypes was a compound heterozygote for one known mutation, c.2289G > T, and another missense variant, c.1670C > G (p.Thr557Ser), with unknown functional impact. This latter variant has not been reported in any other population previously.

**Conclusions:**

The frequency of known mutations in the *LDLR* gene in this cohort of patients was markedly low compared to frequencies reported in other populations. This highlights the likelihood of a complex, polygenic inheritance of FH in Sri Lankan patients, indicating the need for a comprehensive genetic evaluation that includes the screening for mutations in other genes that cause FH, such as *APOB*, *PCSK9*, and *LDLRAP1.*

## Background

Familial hypercholesterolemia (FH) is an inherited disorder of lipoprotein metabolism characterized by elevated low density lipoprotein (LDL) cholesterol in serum, tendon xanthomas and increased risk of premature coronary artery disease (CAD). FH is primarily an autosomal dominant disorder. The frequency of heterozygous FH is about 1 in 500, while homozygous FH is rare in many populations worldwide [1]. Mutations in the genes that encode proteins involved in LDL uptake and catabolism, the LDL-receptor (*LDLR*), apolipoprotein-B (*APOB*), LDL receptor adaptor protein (*LDLRAP1*) and PCSK9 (*PCSK9*) are known to cause FH, resulting from defective LDL uptake and degradation. This leads to elevations in plasma LDL-cholesterol levels, resulting in the hypercholesterolemia phenotype, and manifesting as xanthomas, atherosclerosis, CAD and other cardiovascular diseases. The majority (60–80%) of the patients with FH harbor mutations in the *LDLR* gene, while mutations in the *APOB* and *PCSK9* genes account for a smaller percentage of autosomal dominant FH. Homozygous and compound heterozygous mutations in *LDLRAP1* produce a rare autosomal recessive form of FH [1–3].

The *LDLR* spans 45 kb on the short arm of chromosome 19 and comprises 18 exons that are transcribed and translated into five distinct domains which form the cell surface LDL receptor protein. Mutations in the *LDLR* gene have been classified into 5 groups according to the functional significance of the mutation. Mutations that produce null-alleles with no receptor protein production are classified as ‘class 1’ mutations. The other 4 classes (classes 2–5) include 4 different types of mutations causing a defect in one of the 4 steps in the LDLR uptake and degradation pathway (Class 2: Defects in LDLR transport from the endoplasmic reticulum to the cell surface, Class 3: Defects in the binding of the LDLR to Apolipoprotein B in LDL particles, Class 4: Defects in internalization of the LDL particle-LDLR complex, and Class 5: Defects in recycling of the LDLR protein). Class 1 mutations are known as ‘receptor-negative’ mutations, while the mutations in class 2–5 are commonly described as ‘receptor-defective’ mutations [4, 5]. Association between the different classes of LDLR mutations with the different phenotypic characteristics of FH that include serum LDL-C levels, and clinical manifestations such as tendon xanthomas and risk of CAD have been observed. Patients with receptor negative mutations compared to those with receptor-defective mutations were shown to have higher total cholesterol and LDL-C levels, lower HDL-cholesterol levels and a higher incidence of tendon xanthomas, carotid atherosclerosis and CAD [6–8]. Moreover, receptor-negative mutation carriers had a weaker LDL-cholesterol lowering response to statins [8–10]. These findings highlight the implications of a definitive genetic diagnosis of FH in clinical surveillance, prognostication and management of FH. It is also likely that the carriers of *LDLR* mutations, especially those with ‘receptor defective’ mutations would not develop the stigmata of hypercholesterolemia until the later stages of the disease, making it difficult to diagnose the disease clinically at young age. In such cases with suspected FH, molecular genetic evaluation will be beneficial for confirmation of the disease and to initiate the treatment and follow-up at an earlier age, thus preventing cardiovascular complication.

To date nearly 1500 ‘pathogenic’ and ‘likely pathogenic’ variants of the *LDLR* gene are listed in the ClinVar database. These include a large number of single nucleotide substitutions producing nonsense, missense, frameshift or splice site variants or variants in the promoter or untranslated regions, as well as copy number variants (CNVs) such as duplications, insertions and deletions [11]. It was previously suggested that exons 3 and 4 of the *LDLR*, which correspond to the ligand-binding domain of the LDLR protein, harbor the majority of the mutations in patients with FH. However, the current understanding is that damaging mutations are distributed throughout the gene without clustering in any specific domains of the LDLR protein. [11, 12]. The presence of many different mutations widely spread throughout the *LDLR* gene makes a compelling case for complete sequencing of the *LDLR* coding region in index patients. Identification of a mutation in a patient enables comprehensive genetic counselling. Once an index patient has been genetically diagnosed, cascade screening of the family can identify individuals at risk for increased CAD, and treatment can commence before adulthood. Early treatment, sometimes beginning as early as 9–10 years old, leads to better cardiovascular outcomes in these patients [13].

Despite the extensive heterogeneity of *LDLR* gene reported among patients with FH, some studies have reported the aggregation of certain types of mutations in different geographic areas and different populations. For example, 3 specific *LDLR* mutations, FH-North Karelia, FH-Helsinki, and FH-Turku account for almost 80% of FH cases in the Finnish population [14]. As well, a study in the Netherlands revealed that 4 *LDLR* mutations account for nearly half of the patients with FH [15]. It has been shown that one mutation designated p.Cys681X in the *LDLR* gene accounts for FH in 81.5% of Lebanese FH patients [16] and an “Iceland-specific” splice-site mutation (c.698 + 2T4C [IVS4 + 2T4C]) in the *LDLR* gene was identified in 60% of the patients with FH in Iceland [17, 18]. Geographic clustering of these mutations is probably the result of a founder effect. These findings imply that population-specific frequencies and types of *LDLR* mutations are possible among Sri Lankans. Anecdotal evidence suggests that the incidence of premature CAD is rising in Sri Lanka, and a large number of patients are diagnosed with hypercholesterolemia at a younger age. However, the molecular genetic evaluation of these patients has never been performed in Sri Lanka. Hence our study was designed to assess the frequency and spectrum of *LDLR* mutations in a cohort of patients who were clinically diagnosed to have FH.

## Methods

### Patient recruitment

Patients were selected from consecutive patients seen at the University Medical Unit, University of Colombo, Sri Lanka between October 2011 and March 2012. Patients who were clinically diagnosed with FH were recruited for the study. Diagnosis of FH was performed according to either the Modified Simon Bloom criteria [19] or the Dutch Lipid Clinic Network criteria [20, 21]. Personal and family history of CAD, peripheral vascular disease (PVD), cerebrovascular disease (CVD) and hypercholesterolemia were evaluated and the family data was entered in to a three-generation pedigree chart. Relevant details including the serum lipid levels (i.e. Total cholesterol, LDL-cholesterol and HDL-cholesterol) at the time of initial presentation were obtained from patient’s previous clinic records. All the patients selected for this study were clinically evaluated for peripheral stigmata of hypercholesterolemia (i.e. tendon xanthomas, xanthelasma and corneal arcus). Detailed examination of the cardiovascular and central nervous systems was performed.

### *LDLR* sequencing

Genomic DNA was extracted from peripheral whole blood samples and screened for *LDLR* mutations by standard bi-directional Sanger sequencing. All 18 exons of the *LDLR* gene from all the different transcripts, and approximately 50 bp of flanking intronic sequences at each exon-intron boundary were amplified by polymerase chain reaction. Primer sequences are available upon request. Sequences were analyzed using Sequencher (Genecodes) and compared to the human *LDLR* reference sequence (Genome Reference Consortium Human Build 38 patch release 7 (GRCh38.p7), NM_000527.4). All the identified variants were confirmed by re-sequencing. Written informed consent for the participation in this study and for the genetic testing was obtained from all the participants. The study was approved by the Ethics Review Committee, Faculty of Medicine, University of Colombo, Sri Lanka.

## Results

A total of 27 patients, 13 (48%) males and 14 (52%) females were recruited for the study. The age of the patients ranged between 24 and 73 years (mean 50.1±12.2). Most of the patients (22; 81.5%) did not have any peripheral stigmata of hypercholesterolemia. Xanthelasma was observed in 5 (18.5%) patients and corneal arcus in one (3.7%). None of the patients had tendon xanthoma. The most common phenotype of FH in the study cohort was CAD, which was observed in 10 (37%) patients, 5 of whom had triple vessel disease, and 4 had double vessel disease as determined by coronary angiography, all requiring surgical interventions. One (3.7%) patient had a past history of CVD. None of the patients had peripheral vascular disease (PVD). The majority (24; 88.9%) of the patients had a family history of hypercholesterolemia and/or CAD. Seventeen (63.0%) patients had at least one first degree relative with hypercholesterolemia, and 16 (59.2%) had at least one first degree relative with CAD. In the entire cohort, mean total cholesterol was 356.8 mg/dl (±66.4) and mean LDL-cholesterol was 250.3 mg/dl (±67.7).

When the coding region of the *LDLR* gene was sequenced, pathogenic mutations were detected in 5 (18.5%) patients. Phenotypic characteristics of these mutation-positive patients are summarized in Table 1.

**Table 1:** Phenotypic and genotypic characteristics and the patients with *LDLR* mutations

Patient ID	Age (years)	Sex	Medical history^a^	Family history^b^	Peripheral signs of HC	TC (mg/dl)	LDL-C (mg/dl)	Type of *LDLR* Mutation
S2	52	F	CAD	CAD,HC	XL	503	418	c.1670C > G c.2289G > T^c^
S3	53	F	None	HC,CVA	None	441	344	c.682G > C^d^
S19	39	M	None	HC	None	419	325	c.1855 T > A^d^
S23	43	M	DM,HT	None	None	353	261	c.1720C > A^d^
S26	32	F	None	HC	None	236	151	c.682G > C^d^

^a^Medical problems other than hypercholesterolemia. ^b^Affected first degree relatives. *CAD* coronary heart disease, *CVA* cerebrovascular accident, *DM* diabetes mellitus, *HC* hypercholesterolemia, *HT* hypertension, *LDL-C* LDL cholesterol, *TC* total cholesterol, *XL* xanthelesma

^c^Compound heterozygous mutations/variants, ^d^Heterozygous mutation

**Fig. 1:**
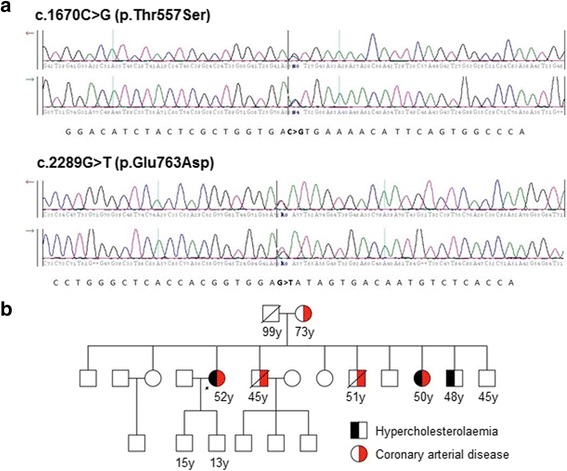
**a**- sequencing results, **b**- Pedigree of the patient with two variants in the *LDLR* gene. Arrow – proband

Four of the 5 patients were heterozygotes for 1 previously identified mutation each. Two of them (mother and son) had the same missense mutation designated c.682G > C (p. Glu228Gln) in exon 4 of the *LDLR* gene. Missense mutations c.1720C > A (p.Arg574Ser) in exon 12 and c.1855 T > A (p.Phe619Leu) in exon 13 were detected in another 2 patients. One patient was a compound heterozygote for a missense mutation c.2289G > T (p.Glu763Asp) in exon 15 and another missense variant, c.1670C > G (p.Thr557Ser) in exon 11 (Fig. 1a). The c.1670C > G is a novel variant that has not been reported in any other population before. This compound heterozygous 52 year old female patient was diagnosed with triple vessel disease by coronary angiography. There is a strong family history of CAD and hypercholesterolemia among several first degree relatives of this patient (Fig. 1b). She had the highest levels of total cholesterol and LDL-cholesterol among the five mutation-positive patients.

The details of the 5 missense mutations detected in the present study are shown in Table 2. We have also identified 14 synonymous variants, and 16 (59.2%) patients were homozygous for the synonymous variant c.1413A > G (p.Arg417Arg).

**Table 2:** Characteristic of the *LDLR* mutations found in the present study

Location	Nucleotide change	Protein change	Allele name	References
Exon 4	c.682G > C	p.Glu228Gln	FH Tulsa 2/ FH Iraq	4,15,23–26
Exon 11	c.1670C > G	p.Thr557Ser	–	Novel variant
Exon 12	c.1720C > A	p.Arg574Ser	–	31
Exon 13	c.1855 T > A	p.Phe619Leu	–	30
Exon 15	c.2289G > T	p.Glu763Asp	–	32

## Discussion

This study is the first to report the mutations in the *LDLR* gene in the Sri Lankan population. Detailed descriptions of the phenotypic characteristics of the Sri Lankan patients with FH are also lacking in scientific literature. Phenotypically, the most notable observation in the present study was the rarity of the peripheral stigmata of hypercholesterolemia in our study cohort. Tendon xanthoma, which is generally considered specific for the FH was reported to be present in 30–60% of patients in Western populations, more commonly among patients with receptor negative mutations [6, 22]. In contrast, tendon xanthomas were not observed in any of the patients in the present study. Statistically significant association between FH and CAD has been demonstrated in studies done in both European and Asian populations [5]. A similar trend was observed in our cohort with a high percentage of patients with CAD requiring surgical interventions. Our results also highlight the value of obtaining detailed family histories of the patients clinically diagnosed with FH.

Four of the five mutations detected in the present study have been previously reported. The missense mutation FH Iraq (c.682G > C), which was detected in 2 related patients in the present study was first reported in 1992 in African Americans [4]. This mutation is in the exon 4 which is within the ligand binding domain (i.e. exon 2–6). The majority of *LDLR* mutations reported to-date are in the exon 4. However, this might have arisen from a selection bias, since many early studies assessed the clinical and functional relevance of mutations in exon 4, and targeted screening of exon 4 in many studies on FH may have contributed to this observation [5]. It has been reported that even a single amino acid substitution in the ligand binding domain of the LDLR protein causes defective binding of the LDLR to apoB-100 apolipoproteins in the LDL particles, thereby impairing the cellular uptake of LDL, and leading to elevations in serum LDL-cholesterol levels [4]. Since the first report of this mutation in African Americans 1992, it has been described in several other populations in Europe [15, 23, 24], Israel [25] and recently, in Japan [26]. Neither of the two patients with this mutation in the present study had a significant medical history other than hypercholesterolemia. However marked differences were observed in the total cholesterol and LDL-cholesterol levels at the initial presentation between these 2 patients with the same familial mutation (S3 and S26 in Table 1). Similarly, intra-familial variations in the clinical expression of FH among patients with the same *LDLR* mutation has been observed in other family-based studies [27, 28]. A case-control study conducted in the French-Canadian population has also suggested gender-specific pleiotropic effects of *LDLR* mutations [29]. Together, this highlights that the phenotypic characteristics of the patients with FH are determined by their genetic as well as other risk factors. This is an important fact that has to be taken into account in counselling mutation-positive patients and in planning their management and follow-up.

The heterozygous missense mutation, c.1855 T > C (p.Phe619Leu) in exon 13 of the *LDLR* gene was detected in one patient in our study, and has been previously reported in a study from the United Kingdom [30]. The missense mutation c.1720C > A (p.R574S) in exon 12 detected in another patient was described recently in a 13-year old Chinese boy with FH. This mutation was predicted to be pathogenic using function prediction bioinformatics tools [31]. However, this mutation is not listed in any of the human genome variant databases.

One of the two variants [c.2289G > T (p.Glu763Asp)] detected in the patient with compound heterozygosity has been previously reported in a study conducted in Western Australia. This missense variant has been classified as a ‘mutation of unknown pathogenicity’ because of the different interpretations of pathogenicity in different function prediction tools [32]. The other variant identified in this compound heterozygote patient, c.1670C > G (p.Thr557Ser), is not listed in any of the human genome/exome variant databases and also has not been described. This variant leads to a conservative change in amino acid from Threonine to Serine at position 557. It is predicted as ‘disease causing variant’ and ‘probably damaging variant’ in MutationTaster [33] and PolyPhen-2 [34] databases respectively, and as ‘neutral/tolerated’ in the PROVEAN/SIFT [35, 36] database. Further in-vitro studies are required to assess the functional impact of this variant. Considering the uncertain pathogenicity of the first mutation, c.2289G > T, in this patient, it is very likely that this second novel mutation has an additive damaging effect, giving rise to the severe phenotypic expression in this patient. This is in concurrence with previous studies that described a more severe clinical and biochemical phenotype associated with biallelic (homozygous or compound heterozygous) FH mutations [1, 5, 32, 37]. Moreover, the diagnosis of compound heterozygosity for *LDLR* mutations has important therapeutic implications. These patients are likely to have poor response to statins, because they are lacking functional LDL receptors in the liver that can be up-regulated by statins [37]. The pathogenicity of the novel variant identified in this study could have been tested by carrying out co-segregation studies looking for the co-inheritance of the variant with elevated LDL-C levels in family members. However, the interpretation of family data may be complicated by the overlay of environmental factors that influence lipid levels and by the presence of other genetic variants that raise or lower LDL-C in the family. Still, we could not perform the segregation studies on this family due to the unapproachability of these family members for testing.

The frequency of *LDLR* mutations in our cohort of patients is very low (18.5%) compared to the population frequency of 60–80% that has been described [1–3]. Possible reasons for this low frequency of *LDLR* mutations include the presence of mutations in other genes that cause FH (i.e. *APOB*, *PCSK9*, and *LDLRAP1*) that were not tested in the present study, and the likelihood of polygenic inheritance of FH in Sri Lankan patients. There may also be other genes associated with FH that have yet to be identified. Mutations in the intronic regions of the *LDLR* gene outside the amplified exonic and flanking regions were not detected by the testing methodology of the present study. Major structural rearrangements of the *LDLR* gene would also have been missed by this technique contributing to the low mutation detection rate. This highlights the need of a comprehensive gene panel that includes the sequencing of the whole *LDLR* gene and other genes with a recognized or a potential role in the pathogenesis of hypercholesterolemia, for genetic diagnosis. Though expensive at present, this method will allow the definite molecular genetic diagnosis of the FH in index patients. The identification of a mutation in index patients enables the cascade screening of family members [38]. It has been shown that cascade screening increases the percentage of patients receiving treatment for FH [39–41] resulting in a significant improvement in the serum lipid parameters following the genetic diagnosis [41, 42]. These studies highlighted the value of cascade screening in early diagnosis and treatment of FH, which will lead to a reduction in mortality and morbidity associated with premature coronary artery disease in these patients.

## Conclusions

This is the first report of the spectrum of *LDLR* variants in a cohort of Sri Lankan patients with FH. We report the discovery of a novel variant and the low contribution of pathogenic variants in the *LDLR* gene to FH in this cohort. This highlights the need for a compressive study involving all the genes implicated in FH to understand the genetic aetiology of the condition in our population.

## Abbreviations

ApoB: Apolipoprotein-B
CAD: Coronary artery disease
CVD: Cerebrovascular disease
FH: Familial hypercholesterolemia
HDL: High-density lipoprotein
LDL: Low-density lipoprotein
LDLR: LDL-receptor
PVD: Peripheral vascular disease
